# Utrecht-Management of Identity Commitments Scale: Validation in Spanish University Students

**DOI:** 10.3389/fpsyg.2018.01364

**Published:** 2018-08-03

**Authors:** Vicente J. Llorent, Mercedes Álamo

**Affiliations:** Faculty of Education, University of Córdoba, Córdoba, Spain

**Keywords:** identity, U-MICS, commitments, exploration, reconsideration of the commitments

## Abstract

The Utrecht-Management of Identity Commitments Scale (U-MICS) has already been validated in nine languages and has had a major scientific impact. Thus, the aim of this study was to validate this instrument in its Spanish version, one of the most important languages in the world. We analyzed the psychometric properties of the scale, applied to a sample of 378 students of the Faculty of Education at the University of Cordoba. The scale consists of 13 items, divided into three dimensions of the process of identity: commitments, in-depth exploration, and reconsideration of the commitments. In this report, the factorial three-dimensional model of process identity is confirmed and shows that the instrument is reliable for measuring the educational, social or global identity of the individual.

## Introduction

Identity development is a process of change that is carried out throughout life, with adolescence serving as one of the most important stages (Erikson, 1950; Schwartz et al., 2011; Zimmermann et al., 2012), since it is the period of transition to adulthood, during which formal operational thought begins and physical, psychosocial, sexual, and cultural growth occurs for the development of autonomy in the individual (Zacarés González et al., 2009; Crocetti et al., 2010; Kłym and Cieciuch, 2015). In adolescence, a process of exploration and decision making on the different alternatives of self-identity begins (Berzonsky et al., 2011).

Erikson’s (1950, 1968) theory of identity argues that, in adolescence, the individual initiates the process of searching for his own identity. From this theory, Marcia (1966) creates a model of categorization of the identity process based on the exploration and commitments of the adolescent (Berzonsky, 2008; Crocetti et al., 2008, 2010, 2012; Zacarés González et al., 2009). Exploration is understood as the active search performed by the individual, and the possible alternatives can be chosen before making any decision based on beliefs, opinions, ideology, or goals. In contrast, the commitments composed of the decisions made at certain points of identity, such as the profession, religion, ideology, etc.

Marcia’s (1966) conceptualizations assume that the individual leaves the exploration stage when he reaches the commitment stage. However, Meeus (1996) further points out that the person can continue to explore after having obtained a firm commitment. For this reason, Luyckx et al. (2006) differentiate between two dimensions: in-breadth and in-depth exploration. These processes are interrelated in identity formation. Therefore, as Meeus (2001, Unpublished) points out, adolescents with firm commitments will continue to explore seriously even though their decisions have already been made. As a result, subsequent studies (Crocetti et al., 2008) have incorporated a new dimension called reconsideration of commitments, and it is in this dimension where the current commitments are compared with other alternatives, which could be more satisfactory for the individual.

In the study of these three dimensions, Meeus developed the Utrecht-Management of Identity Commitments Scale (U-MICS, Crocetti et al., 2008, 2010), reworded from the Utrecht-Groningen Identity Development Scale (Meeus, 1996). The U-MICS evaluates the three dimensions: the commitments, the in-depth exploration and the reconsideration of the commitments. This instrument has been validated with the structure of three factors in different countries, in their respective languages, the Netherlands (Crocetti et al., 2008), Italy (Crocetti et al., 2010), Romania (Negru and Crocetti, 2010), Switzerland (Zimmermann et al., 2012), Turkey (Morsunbul et al., 2014), Poland (Karaś et al., 2015), Bulgaria, Czech Republic, Kosovo, Slovenia (Dimitrova et al., 2015), Portugal, China, Japan, and Taiwan (Crocetti et al., 2015).

Given the large number of studies in different languages for the U-MICS, this research was recommended to validate this instrument in Spanish, and this study aimed to translate and analyze the validity and reliability of the U-MICS in Spanish, one of the most widely used and studied languages in the world, that reaches almost 600 million people, and continues to increase (Instituto Cervantes, 2017).

## Methods

### Participants

The selection of the sample was done incidentally for convenience. The sample is composed of 378 subjects, this being 19.2% of the population of the Faculty of Education Sciences of the University of Córdoba. There are 279 women (73.81%) and 99 men (26.19%), with an average age of 20.96 years old (*SD* = 3.46), with 92.1% of the sample between 18 and 24 years old. The distribution by gender, although not homogeneous, is representative of the population that is being investigated, since the predominance of the female gender is a characteristic of education faculties.

### Measurement Instrument

The U-MICS (Crocetti et al., 2008, 2010) is a scale composed of 13 items, of which five items measure commitments, five items measure in-depth exploration and the last three assess the reconsideration of commitments. The scale is Likert-type (1 totally disagree – 5 totally agree), in which the degree of agreement that the participants have in a domain of the identity is measured as educational, social, or labor. In this research, the first two domains (educational and relational) have been studied (see Appendix). Internal consistency for the factor compromises and reconsideration of the commitments is generally very high with a Cronbach’s alpha (α) over 0.80 and acceptable for in-depth exploration, with a α > 0.70 (Guilford, 1954; Schmitt, 1996; Orozco et al., 2002).

### Procedure

Firstly, the U-MICS has been translated from English to Spanish and again from Spanish into English, to correctly set the meaning of the items. This process has been supervised by three professionals, being one an author with previous experience with this same instrument (Severino et al., 2014).

The next phase was the collection of data, obtaining the collaboration of the faculty for access to the sample. All participants were previously informed of the objective of the investigation and requested anonymity in the completion of the form. The implementation of the instrument lasted 10 min for each class group.

The data was analyzed after it was obtained. First, a descriptive study of all the items was carried out, in which the mean, standard deviation, kurtosis, and asymmetry were analyzed. The internal consistency of the scale was calculated through Cronbach’s alpha.

Given that it is a scale that has been confirmed in different languages and that the dimensions are clear and defined, it has proceeded to its confirmatory factor analysis (CFA) was performed using the maximum likelihood parameter and the following adjustment indexes were used: the comparative fit index (CFI), the non-normalized fit index (NNFI), the goodness index of (GFI), the root mean squared error of approximation (RMSEA) and the standardized root mean square residual (SRMR). The composite reliability coefficient (CR) and average variance extracted (AVE) were also calculated. Finally, the Pearson correlation was performed to determine the relationships between the different dimensions of the scale. For the statistical analyses, EQS 6.2 and SPSS 23 software were used.

## Results

The psychometric properties of the instrument were analyzed for the validation of this instrument in Spanish by studying the reliability and validity of each construct.

Initially, the analysis of global internal consistency was performed using the Cronbach’s alpha coefficient. The result obtained for the educational domain has been for the 13 items a value of α = 0.80 and for the social domain scale of the 13 items was a value of α = 0.80, showing a high internal reliability in both cases. **Table 1** presents the results of the internal consistency disaggregated in each latent factor. Both domains of identity (educational and relational) obtained similar results in internal consistency. The results of the commitments dimension showed excellent reliability in the social domain, and in the educational domain is approaching 0.90, which is a good result for this dimension. The dimension of reconsideration of the commitments also showed a good reliability, surpassing 0.80 in both domains. Finally, the in-depth exploration presents an acceptable reliability, surpassing 0.70 (George and Mallery, 2003).

**Table 1:** Descriptive features and analysis of the internal consistency of the dimensions of the Utrecht-Management of Identity Commitments Scale (U-MICS).

Dimension	School domains						Relational domains/Best friend					
	α	Item	*M*	*SD*	*As*	*K*	α	Item	*M*	*SD*	*As*	*K*
Commitment	0.88	SI1	4.31	0.87	-1.34	1.87	0.93	IS1	3.83	1.06	-0.73	-0.00
		SI2	4.27	0.87	-1.38	2.25		IS2	3.93	1.04	-0.95	0.46
		SI3	4.28	0.83	-1.30	2.08		IS3	3.88	1.09	-0.93	0.30
		SI4	4.20	0.96	-1.09	0.59		IS4	3.45	1.15	-0.40	-0.55
		SI5	3.96	0.99	-0.74	0.02		IS5	3.73	1.09	-0.71	-0.04
In-depth exploration	0.74	SI6	3.81	0.89	-0.52	0.12	0.79	IS6	4.11	0.96	-1.11	1.04
		SI7	3.73	1.04	-0.68	0.14		IS7	3.56	1.12	-0.42	-0.54
		SI8	4.11	0.88	-0.92	0.68		IS8	3.86	0.99	-0.68	0.06
		SI9	3.23	1.15	-0.09	-0.80		IS9	3.16	1.24	-0.14	-0.99
		SI10	3.20	1.16	-0.13	-0.76		IS10	3.45	1.19	-0.40	-0.66
Reconsideration of commitment	0.87	SI11	2.89	1.26	0.05	-1.03	0.86	IS11	1.78	1.23	1.38	0.60
		SI12	2.81	1.33	0.19	-1.12		IS12	1.93	1.17	1.04	0.00
		SI13	2.20	1.26	0.69	-0.71		IS13	1.56	1.05	1.91	2.81


To confirm the two domains (educational and relational) of the U-MICS scale used for this study, a confirmatory factorial analysis (AFC) is performed with the maximum likelihood method with the statistical program EQS 6.2.

Following the theoretical approaches, four structural models were performed on the scale. The first one consists of a single factor, in which all the items load in a single dimension of the identity. The second model sets out two factors: commitments and global exploration (consisting of in-depth exploration and reconsideration of jointly grouped commitments). The three-factor model is composed of commitments, in-depth exploration, and reconsideration of commitments. The last model is the same three-factor structure with corrections of three correlated errors.

**FIGURE 1:**
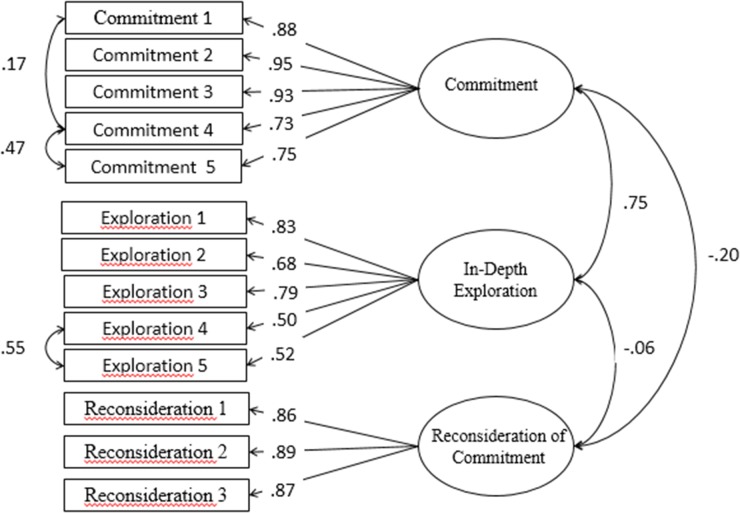
Structural model with three factors and three correlated errors of the Utrecht-Management of Identity Commitments Scale (U-MICS).

As shown the **Table 2**, one-factor and two-factor models do not show any adjustment in educational identity or relational identity. The three-factor model is based on the Crocetti et al. (2008) study, which achieves an acceptable fit on the social identity scale for the CFI and NNFI indexes. Despite these results, it was still necessary to adjust the model. Thus, a correlation with three errors in the three-factor model (Severino et al., 2014; Crocetti et al., 2015) was performed, giving high scores on the adjustment in all indexes analyzed in both scales (see **Figure 1**).

**Table 2:** Structural models of the U-MICS on the basis of a confirmatory factor analysis.

Models	χ^2^	*df*	χ^2^/*df*	CFI	NNFI	GFI	SRMR	RMSEA
**School identity**								
1 Factor	1003.347	65	15.43	0.53	0.44	0.70	0.16	0.19
2 Factors	598.513	64	9.35	0.73	0.67	0.76	0.17	0.15
3 Factors	291.914	62	4.70	0.88	0.85	0.89	0.08	0.10
3 Factors with 3 correlated errors	170.230	59	2.88	0.94	0.93	0.93	0.06	0.07
**Relational identity/Best friend**								
1 Factor	957.437	65	14.72	0.68	0.61	0.68	0.14	0.19
2 Factors	793.147	64	12.39	0.73	0.68	0.71	0.13	0.17
3 Factors	271.887	62	4.38	0.92	0.90	0.89	0.07	0.09
3 Factors with 3 correlated errors	208.103	59	3.52	0.94	0.92	0.91	0.06	0.08


**Table 3** shows the composite reliability (CR) of the three dimensions in their two domains, where compromises and reconsideration obtained high values, since they exceeded 0.80. In addition, the mean extracted variance (AVE) was calculated, where the commitment and reconsideration dimensions also exceeded 0.50.

**Table 3:** Composite reliability coefficients and average variance extracted of the U-MICS.

Domains	School identity		Relational identity/Best friend	
Dimension	*CR*	*AVE*	*CR*	*AVE*
Commitment	0.80	0.50	0.89	0.65
In-depth exploration	0.61	0.27	0.78	0.42
Reconsideration of commitment	0.88	0.72	0.86	0.68


Pearson’s correlation analysis was also performed to measure the relationship between the latent variables of the study. The results of the educational identity showed a relationship between commitments with in-depth exploration (*r* = 0.294, *p* < 0.01) and reconsideration of the commitments with in-depth exploration (*r* = 0.252, *p* < 0.01). Relationships between dimensions have been positive and low for the domain of educational identity.

On the other hand, a relationship has been found in the social identity domain between commitments with in-depth exploration (*r* = 0.529, *p* < 0.01) and commitments with reconsideration of commitments (*r* = -0.264, *p* < 0.01). In this case, a moderate correlation has been found (compromises with in-depth exploration). Correlation was low and negative.

## Discussion

The goal of validation of the U-MICS identity scale in its educational and relational domain, proposed by Meeus (2001, Unpublished), has been accomplished. Thus, it is possible to have this instrument validated in Spanish and added to the list of other languages, to which the scale has been translated (Crocetti et al., 2008, 2010, 2015; Dimitrova et al., 2015). This article facilitated application of the U-MICS to a wide geographic and population area in order to continue the studies on identity.

The results denote a model composed of three factors with three correlated errors, confirming the results of other investigations (Crocetti et al., 2008, 2011; Zimmermann et al., 2012). The three factors are “Commitments,” “In-depth exploration,” and “Reconsideration of commitments” as proposed in the rationale (Crocetti et al., 2008).

Correlation analyses indicate that there is a relationship between the three dimensions. In the educational domain, commitments to deep exploration are positively correlated (Crocetti et al., 2008, 2010; Zimmermann et al., 2012), as is the dimension of the reconsideration of the commitments to in-depth exploration (Crocetti et al., 2008, 2010). In the social domain, the existing relationships were positive between commitments to reconsider commitments (Crocetti et al., 2008, 2010; Zimmermann et al., 2012) and negative between commitments with reconsideration of commitments (Zimmermann et al., 2012).

## Conclusion

In conclusion, this scale, in its educational and social domain, obtains good preliminary results, proving that it is a reliable instrument for measuring identity. It is proposed, as the beginning of another study, to validate the domain of labor identity in the Spanish version, to make a comparison of the three identity domains and to evaluate the possibility of studying the extent to which training favors the construction of identity in different educational levels.

## Ethics Statement

The approval of the ethics commission of the University of Córdoba will be requested and obtained before carrying out the present study. It is a research through a survey therefore, according to national, international, and University of Córdoba regulations, obtaining such approval is voluntary. As stated in the legislation, information will be provided to all participants. Researchers will not exert any pressure on students to get their participation in the study. This work will comply with international and Spanish legislation and with international reference documents, such as the Declaration of Helsinki. The participation will be voluntary and totally anonymous, complying with the data protection law. The benefits of this research will be shared with local, national, and international actors.

## Author Contributions

VL and MÁ contributed to conception and design of the study, performed the statistical analysis, contributed to manuscript revision, and read and approved the submitted version. MÁ organized the database and wrote the first draft of the manuscript.

## Conflict of Interest Statement

The authors declare that the research was conducted in the absence of any commercial or financial relationships that could be construed as a potential conflict of interest.

## Supplementary Material

The Supplementary Material for this article can be found online at: https://www.frontiersin.org/articles/10.3389/fpsyg.2018.01364/full#supplementary-material
