# Supplementary material for: Utrecht-Management of Identity Commitments Scale: Validation in Spanish University Students
Source: Front Psychol. 2018 Aug 3;9:1364. doi: 10.3389/fpsyg.2018.01364 (PMC6085597; doi:10.3389/fpsyg.2018.01364)
Supplement: Supplementary file 1 [file Data_Sheet_1.DOCX]

**Appendix**

**The Utrecht-Management of Identity Commitments Scale (Versión Española)**

**School Identity**

Debajo hay una serie de cuestiones sobre tu educación escolar. En cada caso, coloca una cruz en la casilla que mejor se corresponda con tu opinión, donde 1 es "Totalmente en desacuerdo y 5 "Totalmente de acuerdo".

**Commitment**

1. Mi educación me proporciona seguridad en la vida
2. Mi educación me da confianza en mí mismo
3. Mi educación me hace sentir seguro de mí mismo
4. Mi educación me da seguridad en mi futuro
5. Mi educación me permite afrontar el futuro con optimismo

**In-Depth Exploration**

1. Intento conocer muchos detalles sobre mi educación
2. A menudo reflexiono sobre mi educación
3. Hago un gran esfuerzo para seguir aprendiendo cosas nuevas
4. A menudo intento descubrir lo que otras personas piensan sobre mi educación
5. A menudo hablo con otras personas de mi educación

**Reconsideration of Commitment**

1. A menudo creo que sería mejor intentar encontrar una educación diferente
2. A menudo creo que una educación diferente haría mi vida más interesante
3. A decir verdad, estoy buscando una educación diferente

**Relational Identity/ Best Friend**

Debajo hay una serie de preguntas sobre tu mejor amigo. Nota: con “tu mejor amigo” no nos referimos a un hermano, o alguien con quien estés saliendo. En cada caso, coloca una cruz en la casilla que mejor se corresponda con tu opinión, donde 1 es "Totalmente en desacuerdo y 5 "Totalmente de acuerdo".

**Commitment**

1. Mi mejor amigo me da seguridad en la vida
2. Mi mejor amigo me da confianza en mí mismo
3. Mi mejor amigo me hace sentirme seguro de mí mismo
4. Mi mejor amigo me da seguridad para el futuro
5. Mi mejor amigo me permite afrontar el futuro con optimismo

**In-Depth Exploration**

1. Intento conocer muchos detalles sobre mi mejor amigo
2. A menudo reflexiono sobre mi mejor amigo
3. Hago un gran esfuerzo para seguir conociendo cosas nuevas sobre mi mejor amigo
4. A menudo intento descubrir lo que otras personas piensan de mi mejor amigo
5. A menudo hablo con otras personas de mi mejor amigo

**Reconsideration of Commitment**

1. A menudo creo que sería mejor intentar encontrar otro mejor amigo
2. A menudo creo que un nuevo mejor amigo haría mi vida más interesante
3. A decir verdad, estoy buscando un nuevo mejor amigo
